# Sensitization to Common and Uncommon Pets or Other Furry Animals: Which May Be Common Mechanisms?

**Published:** 2016-05-16

**Authors:** G Liccardi, M Triggiani, A Piccolo, A Salzillo, R Parente, F Manzi, A Vatrella

**Affiliations:** 1 Department of Chest Diseases, Division of Pneumology and Allergology. High Speciality “A.Cardarelli” Hospital, Naples Italy; 2 Division of Allergy and Clinical Immunology, Department of Medicine and Surgery, University of Salerno, Italy; 3 Division of Respiratory Disease, Department of Medicine and Surgery, University of Salerno, Italy

**Keywords:** Allergic rhinitis, allergic sensitization, bronchial asthma, cat, dog, pet allergy

## Abstract

Exposure to animal allergens constitutes a relevant risk factor for the development of allergic sensitization. Moreover, an increasing number of people become owners of less common animals. In this article we summarize aspects related to sensitization to cat/dog which may be applied also to uncommon pets or other furry animals.

The data discussed here suggest that several different factors may induce allergic sensitization to furry animals with or without previous contact. Allergic sensitization without animal exposure is a relevant risk for patients because they are not aware about the possibility that even severe respiratory symptoms may develop after an occasional animal contact. This aspect should be taken into account by susceptible individuals before acquiring pets or beginning a contact for working/leisure activity with a common as well as uncommon animal. As a consequence, skin prick test and/or evaluation of specific IgE antibodies (by classic ImmunoCAP or micro-array technique ImmunoCAP ISAC) also to less common (“new”) mammalian allergens could be recommended in individuals already sensitized to common pets to identify the occurrence of allergic sensitization and consequently to avoid future exposures to uncommon animal allergens.

## I. INTRODUCTION

Exposure to animal allergens constitutes a relevant risk factor for the development of allergic sensitization and respiratory allergic diseases, such as asthma and rhino-conjunctivitis in susceptible individuals [1, 2]. Cats and dogs are the most common pets living indoor environments and the frequency of their ownership is highly variable in Europe ranging from 7.2 to 35% for the cats and from 5.4 to 35 % for the dogs [3].

The prevalence of allergic sensitization to cats /dogs varies in different countries according to cultural differences, environmental factors and rate of pet ownership. Recent studies have shown that the prevalence of sensitization is increasing over the past decades [4,5]. Available epidemiological data are based on some large studies carried out in Unites States and Europe, other data obtained from single countries are less significant as it characterized by lower numbers of patients. In Europe, the cat sensitization rate was 26.3% ranging from 16.8 to 49.3% and the dog sensitization rate was 27.2% ranging from 16.1 to 56%. The prevalence of sensitization to both pets was particularly high in some Northern European countries (e.g. Denmark and Finland) and lower in Central/Western and in Mediterranean countries (e.g. Belgium, Austria and Italy) [6]. In US, allergic sensitization rate among children suffering from asthma was 41% for cat and 21% for dog allergens [7].

Allergy to common pets (or other furry animals) may also occur in some occupational settings where workers are intensively exposed to animal dander during most of their working time [8]. In all developed countries, an increasing number of people become owners of less common animals, especially mammals [9–11]. Although several allergens from these animals have been identified, few data are available on epidemiology, characteristics of exposure and sensitization to these allergens.

In this article we describe some issues on allergy to uncommon pets and summarize some important aspects related to sensitization to cat/dog which may be applied also to uncommon pets or other furry animals.

## II. MECHANISMS AND FACTORS RELATED TO ALLERGIC SENSITIZATION TO ANIMAL ALLERGENS

**Figure 1.**
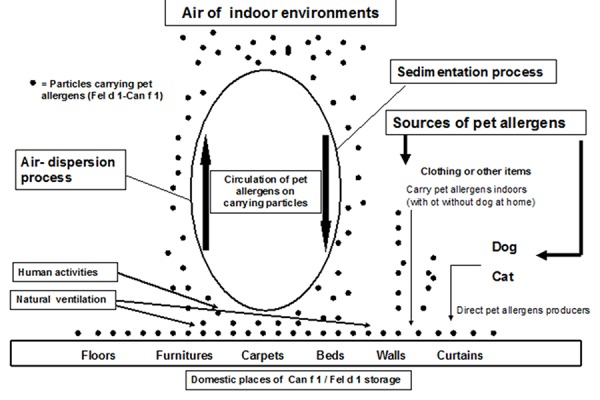


Cat and dog allergens should be considered as ubiquitous because they are found not only in indoor environments where these animals are kept, but also in other indoor private or public places where cats/dogs have been never kept [12] (Table 1). Dynamics of cat /dog allergens in indoor environments are complex because the amounts of these allergens found in reservoirs depend from the presence of animals at home as well as from passive transport of allergens indoors (Figure 1). It is essential to underline that the percentage of pet allergens carried on small particles (about 0.5–2 µm) become easily airborne under normal domestic ventilation and it constitutes the main material able to trigger respiratory symptoms in sensitized patients.

These allergens are also able to reach lower airways inducing prolonged bronchial obstruction [13].

**Figure 2.**
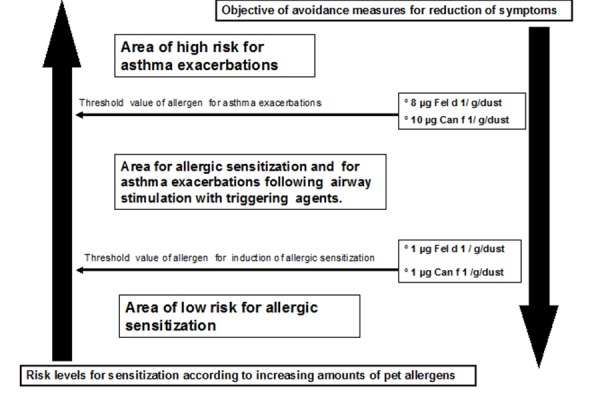


Accumulation of pet allergens in indoor environments without animals has been demonstrated to correlate with the number of visitors owning a pet or with those who are in regular contact with these animals. Therefore, the higher the pet ownership in a given community the higher the presence of pet allergens in apparently pet-free spaces [14]. We and others have shown that clothing and other items, such as human hair, constitute a means for transferring pet allergens in pet-free indoor environments [15,16] (Figure 1). These indoor environments contaminated by pet allergens are able to induce allergic sensitization in susceptible individuals and trigger respiratory symptoms in already highly sensitized subjects [17,18]. In fact, in these contaminated environments, especially schools, the amount of pet allergens is higher than threshold values generally recognized as sufficient to induce sensitization or trigger respiratory symptoms, i.e.,1 µg and 8–10 µg of allergen/g of dust respectively [19] (Figure 2).

In developed countries, the consequence of pet allergen ubiquity is a persistent stimulation of airways similar to that induced by dust mite, that would increase the risk of allergic sensitization either directly or by a cross-reaction mechanism involving albumins and lipocalins [20,21].

Lipocalins constitute the most important group of mammalian inhalant allergens because they are the major allergenic materials derived from dog (Can f 1–2), cattle (Bos d 2), horse (Equ c 1), rat (Rat n 1), mouse (Mus m 1), guinea pig (Cav p 1), rabbit (Ory c 1), hamster (Pho s 21) [2]. The role of lipocalins is to carry small hydrophobic molecules and pherormones.

Serum albumins (SAs) constitute the major component of proteins in the circulatory system of mammals, their functional role is the control of colloid osmotic blood pressure and the transport of ligands. The molecular weight of serum albumins is 66–69 kDa [20]. It has been shown that mammalian serum albumins exhibit a very high amino-acid identity to human serum albumins (about 72–82 %) [22].

SAs represent a group of minor allergens in mammals, in some cases they have been well identified such as Fel d 2 (in cat), Can f 3 (in dog), Equ c 3 (in horse) but SAs have been found as sensitizing agents also in rat, mouse and rabbit. It is likely that SAs play a significant role, as cross-reacting allergens in individuals sensitized to several animal dander in association with lipocalins and other environmental conditions. These mechanisms could explain the peculiarity of some atopic patients to develop allergic sensitization to mammalian allergens also in the absence of contact with the animals. We reported an unusual case of a young women suffering from respiratory allergy and showing cutaneous / serological sensitization exclusively to mammalian dander from cat, dog, rabbit, horse, rat, mouse, guinea pig, cow and hamster. Immunoblot analysis revealed IgE - reactivity to cow’s, rabbit’s and horse’s SAs, with a good correlation between intensity of response in SPT (wheal diameter) and the densitometric class of reaction in immunoblotting [23].

SAs are a minor allergen in animal dander and in milk but a major allergen in meats. In view of the reported cross-reactivity between SAs from mammals, even if they are phylogenetically distinct, the diagnostic workup in meat allergic subjects should always include tests with meats from different mammals, and the use of alternative meats should be always carefully evaluated on an individual basis. Further, due to cross-reactivity phenomena, children with persistent milk allergy and bovine serum albumin (BSA) sensitization show an increased risk of clinically relevant sensitization to animal dander [24].

Recently BSA has been added in culture medium of spermatozoids used for artificial insemination. As a consequence some case reports have shown that BSA may be a causative agent in severe anaphylaxis after standard intrauterine insemination or in vitro fertilization [25,26]. These studies reported that the reaction to BSA could be caused by cross-reaction with SAs contained in heterologous allergenic sources. Since it has been demonstrated a relationship between SAs and allergic sensitization to mammalian dander, it is likely that a previous sensitization to animal allergens constitutes a significant risk factor for anaphylaxis in women undergoing these procedures. The different allergenic molecules of cat, dog and other mammals are summarised in Table 2 [2,27].

**Figure 3.**
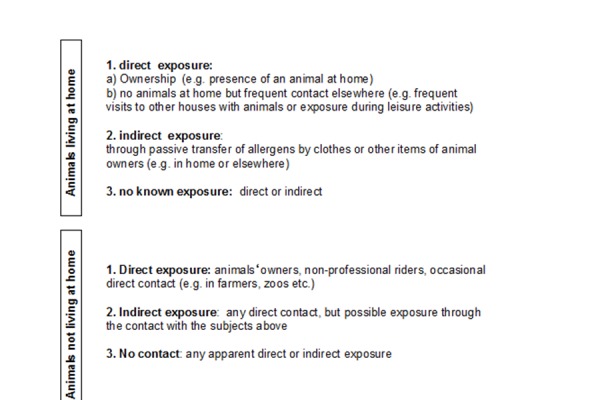


We investigated the role of distinct modalities of exposure to animals in sensitized individuals living in urban areas of Naples and Italy and non-occupationally exposed to any animal. Urban area represents a good model to study all possible modalities of exposure to different animals because the population is not extensively exposed. In this context, we classified three modalities of animal exposure (Figure 3).

In Naples area, only about fifty percent of atopic patients sensitized to common pets (cats/dogs) are directly exposed to these animals, whereas the other half are indirectly exposed or not exposed. If we consider allergic sensitization and modalities of exposure to other furry animals such as rabbits, hamsters, rats, horses, cows, guinea pigs and mouse the percentage of sensitized individuals exposed directly to these animals ranges between 0–33.3 % whereas patients sensitized to the same animals with indirect or no contact ranges between 66.7–100 % [28].

Data on the prevalence of allergic sensitization to some animals such as rabbits and horses in non-occupational settings are very few worldwide, although rabbits are becoming very popular as pets and horses are involved in several leisure activities. We have shown that in Naples area and in Italy the values of prevalence of allergic sensitization to rabbit allergens range between 2.65–4.9 % and 0.65–4.72 % respectively [29–31]. The prevalence of allergic sensitization to horse allergens was 3.43% in Naples area and a mean value of 5.38% (ranging between 2.66–13.46%) in Italy [32,33]. Moreover, the prevalence of sensitization to horse in Italian children has been estimated around 2.7% [34]. With the exception of US, very few data have been published on rodent allergy in other parts of the world including Europe. On behalf of Italian Association of Hospital and Territorial Allergists-Immunologists (AAITO-Campania), we have recently shown that the prevalence of allergic sensitization to mouse and rat allergens is not negligible in urban atopic population living in Campania district area, with a mean value of 3.86% (range 0.72–13%) [35]. This rate is higher in comparison to that found in Naples area previously (1.60% for mouse and 0.59% for rat allergens), this finding is probably due to higher exposure to rodents allergens in other cities of Campania Districts [36]. In both studies we found also a high rate of allergic sensitization also to cat /dog allergens suggesting the possibility of a cross-reacting mechanism.

Finally, we have shown, using *in vivo* model (skin prick test), that exposure and allergic sensitization to common pets increases by about fourteen-fold the risk of developing sensitization to other furry animals suggesting a possible predisposition to develop multiple sensitization to animal allergens [37].

Recently, we have confirmed these finding also using an *in vitro* model (the micro-array technique ImmunoCAP ISAC, Thermofisher Scientific - Immuno-Diagnostics, Sweden, in 741 subjects referred to the Allergy Unit of Fondazione Salvatore Maugeri, Pavia). These *in vitro* data suggest that allergic sensitization to common pets increases the risk of sensitization to horse and mouse because of the presence of lipocalins. Since lipocalins show a certain degree of cross-reactivity, a similar finding for other furry animals is likely [38].

Recently, the role of concomitant exposure from other allergens or microrganisms, the significance of the community prevalence of furry animals as well as the impact of molecular-based allergy diagnostics in the development to allergy to furry animals has been reviewed extensively [39].

Avoidance measures to prevent allergic sensitization to pet allergens are difficult to perform considering the ubiquity of pet allergens and the frequent rejection of pet owners to relocate their animals [40]. Allergen immunotherapy for pet allergens is less used in comparison to other environmental agents probably because the necessity to utilize better purified allergenic materials [41].

## III. CONCLUSION

Currently available data indicate that sensitization to uncommon pets is an emerging problem affecting allergic patients. The data discussed here suggest that several different factors may induce allergic sensitization to furry animals with or without previous contact. Allergic sensitization without animal exposure is a potential risk for patients with rhinconjunctivitis or asthma because they are often unaware that even severe respiratory symptoms may develop after an occasional animal contact. This aspect should be taken into account by susceptible individuals before acquiring pets or beginning a contact for working/leisure activity with common as well as uncommon animals. Skin prick test and/or evaluation of specific IgE antibodies also to “new” mammalian allergens should be recommended in individuals already sensitized to common pets to identify the occurrence of allergic sensitization and consequently to avoid future exposures to uncommon animal allergens [42,43]. In this context an evaluation of specific IgE by using the micro-array technique for lipocalins (Can f 1, Can f 2, Equ c 1, Fel d 4, Mus m 1) and albumins (Bos d 6, Can f 3, Equ c 3, Fel d 2) might be very useful to evaluate the possibility of cross-reactions between allergens of different animals [38,44].

## Figures and Tables

**TABLE I.** LEVELS OF PET ALLERGENS IN ENVIRONMENTS

Environments examined	Levels of pet allergens (*Fel d 1* e *Can f 1*) Range of values expressed as *μg*/g of dust	
	Dog allergens	Cat allergens
Private homes	1.37 – 2.6	0.06 – 61.9
Nurseries	0.2 – 1.8	0.9 – 3.7
Public space (e.g. offices, hospitals, hotels)	0.2 – 63	4.5 – 58.1
Transportation (e.g. buses, cars)	2 – 8.5	0.3 – 2.6
Schools (indoors)	0.2 – 12	0.3 – 118.3
Schools (outdoors)	--	3.18 – 10.8

**TABLE II.** MAINLY MOLECULAR ALLERGENS OF CAT, DOG AND OTHER MAMMALS

**Animal**	**Molecular allergens**	**Family groups**
**Dog**	Can f1	Lipocalin
	Can f2	Lipocalin
	Can f2	Albumin
	Can f4	Lipocalin
	Can f5	Kallicrein
**Cat**	Fel d1	Uteroglobin
	Fel d2	Serum albumin
	Fel d3	Cystatin
	Fel d4	Lipocalin
	Fel d5	Cat IgA
**Rabbit**	Ory c1	Lipocalin
	Ory c3	Lipophilin
**Guinea pig**	Cav p1	Lipocalin
	Cav p2	Lipocalin
	Cav p3	Lipocalin
	Cav p4	Serum albumin
**Mouse**	Mus m1	Lipocalin
	Mus m4	Serum albumin
**Rat**	Rat n1	Lipocalin
	Rat n4	Serum albumin
**Hamster**	Pho s1	Lipocalin
**Pig**	Sus s5	Lipocalin
	Sus s6	Serum albumin
**Horse**	Equ c1	Lipocalin
	Equ c2	Lipocalin
	Equ c3	Serum albumin
**Cattle**	Bos d2	Lipocalin
	Bos d6	Serum albumin

## References

[1] Liccardi G, D’Amato G, D’Amato L, Salzillo A, Piccolo A, De Napoli I, Dente B, Cazzola M (2005). The effect of pet ownership on the risk of allergic sensitization and bronchial asthma. Respir Med.

[2] Hentges F, Leonard C, Arumugan K, Hilger C (2014). Immune response to mammalian allergens. Front Immunol.

[3] Eller E, Roll S, Chen CM, Herbarth O, Wichman HE, Berg A (2008). Meta-analysis of determinants for pet ownership in 12 European birth cohort on asthma and allergies: a GA2LEN initiative. Allergy.

[4] Asher MI, Montefort S, Bjorksten B, Lai CK, Strachan DP, Weiland SK (2006). Worldwide time trends in the prevalence of symptoms of asthma, allergic rhinoconjunctivitis and eczema in childhood: ISAAC Phases One and Three repeat multicountry cross-sectional survey. Lancet.

[5] Arbes SJ, Gergen PJ, Elliott L, Zeldin DC (2005). Prevalence of skin prick test responses to 10 common allergens in the US population: results from the third National Health and Nutrition Examination Survey. J Allergy Clin Immunol.

[6] Heinzerling LM, Burbach GJ, Edenharten G, Bachert C, Bindslev-jensen C, Bonini S (2009). GA2LEN harmonization of skin prick testing: novel sensitization patterns for inhalant allergens in Europe. Allergy.

[7] Gruchalla RS, Pougracic J, Plant M, Evans R, Visness CM, Walter M, Crain E, Kattan M, Morgan WJ, Steinbach S (2005). Inner City Asthma Study: Relationship among sensitivity, allergen exposure, and asthma morbidity. J Allergy Clin Immunol.

[8] Liccardi G, Steinhilber G, Meriggi A, D’Amato G (2014). Sensitization to pets in pet shop workers. Occup Med (Lond).

[9] Phillips JF, Lockey RF (2009). Exotic pet allergy. J Allergy Clin Immunol.

[10] Pauli G, Bessot JC (2009). Rare indoor allergens. Eur Ann Allergy Clin Immunol.

[11] Diaz-Perales A, Gonzalez-de-Olano D, Perez-Gordo M, Pastor-Vargas C (2013). Allergy to uncommon pets: new allergies but the same allergens. Front Immunol.

[12] Liccardi G, D’Amato G, Russo M, Canonica GW, D’Amato L, Passalacqua G (2003). Focus on cat allergen (Fel d 1): immunological and aerodynamic characteristics, modality of airway sensitization and avoidance strategies. Int Arch Allergy Immunol.

[13] Zeidler MR, Goldin JG, Kleerup EC, Kim HJ, Truong DA, Gjertson DW, Kennedy NJ, Newman KB, Tashkin DP, Silverman JM, Corren J (2006). Small airways response to naturalistic cat allergen exposure in subjects with asthma. J Allergy Clin Immunol.

[14] Heinrich J, Bedana GB, Zock JP (2006). For The Indoor Working Group of The European Community Health Survey II. Cat allergen level: its determinants and relationship to specific IgE to cat across European centers. J Allergy Clin Immunol.

[15] D’Amato G, Liccardi G, Russo M, Barber D, D’Amato M, Carreira J (1997). Clothing is a carrier of cat allergens. J Allergy Clin Immunol.

[16] Liccardi G, Barber D, Russo M, D’Amato M, D’Amato G (2005). Human hair: an unexpected source of cat allergen exposure. Int Arch Allergy Immunol.

[17] Bollinger ME, Eggleston PA, Flanagan E, Wood RA (1996). Cat antigen in homes with and without cats may induce allergic symptoms. J Allergy Clin Immunol.

[18] Munir AKM, Einarsson R, Schou C, Dreborg SKG (1993). Allergens in school dust.I. The amount of the major cat (*Fel d* 1) and dog (*Can f* 1) allergens in dust from Swedish schools is high enough to probably cause perennial symptoms in most children with asthma who are sensitized to cat and dog. J Allergy Clin Immunol.

[19] Chapman MD, Wood RA (2001). The role and remediation of animal allergens in allergic diseases. J Allergy Clin Immunol.

[20] Liccardi G, Asero R, D’Amato M, D’Amato G (2011). Role of sensitization to mammalian serum albumin in allergic disease. Curr Allergy Asthma Rep.

[21] Nordlund B, Konradsen JR, Kull I, Borres MP, Onell A, Hedlin G, Gronlund H (2012). IgE antibodies to animal-derived lipocalin, kallicrein and secretoglobulin are markers of bronchial inflammation in severe childhood asthma. Allergy.

[22] Chruszcz M, Mikolajczak K, Mauk N, Majorek KA, Porebski PJ, Minor W (2013). Serum albumins-unusual allergens. Biochem Biophys Acta.

[23] Liccardi G, Dente B, Restani P, Senna GE, Falagiani P, Ballabio C, D’Amato G (2010). Respiratory allergy induced by exclusive poly-sensitization to serum albumins of furry animals. Eur Ann Allergy Clin Immunol.

[24] Vicente-Serrano J, Caballero ML, Rodriguez-Perez R, Carretero R, Blanco JG, Juste S, Moneo I (2007). Sensitization to serum albumins in children allergic to cow’s milk and epithelia. Pediatr Allergy Immunol.

[25] Pagan JA, Postigo I, Rodriguez-Pacheco JA, Pena M, Guisantes JA, Martinez J (2008). Bovine serum albumin contained in culture medium used in artificial insemination is an important anaphylaxis risk factor. Fertil Steril.

[26] Orta M, Ordoqui E, Aranzabal A, Fernandez C, Bartolomè C, Sanz ML (2003). Anaphylactic reaction after artificial insemination. Ann Allergy Asthma Immunol.

[27] Konradsen JR, Fujisawa T, van Hage M, Hedlin G, Hilger C, Kleine-Tebbe J, Matsui EC, Roberts G, Ronmark E, Platts-Mills T (2015). Allergy to furry animals: New insights, diagnostic approach and challenges. J Allergy Clin Immunol.

[28] Liccardi G, Salzillo A, Piccolo A, Russo M, D’Amato G (2011). Sensitization to furry animals in an urban atopic population living in Naples, Italy. Allergy.

[29] Liccardi G, D’Amato G, Canonica GW, Dente B, Passalacqua G (2004). Severe respiratory allergy induced by indirect exposure to rabbit dander: a case report. Allergy.

[30] Liccardi G, Piccolo A, Dente B, Salzillo A, Gilder JA, Russo M, D’Amato G (2007). Rabbit allergens: a significant risk for allergic sensitization in subjects without occupational exposure. Respir Med.

[31] Liccardi G, Passalacqua G, on behalf of the Allergy Study Group of the Italian Society of Respiratory Medicine (SIMeR) (2006). Sensitization to rabbit allergens in Italy- A multicentre study in atopic subjects without occupational exposure. Int Arch Allergy Immunol.

[32] Liccardi G, Salzillo A, Dente B, Piccolo A, Lobefalo G, Russo M, Gilder JA, D’Amato G (2009). Horse allergens: an underestimated risk for allergic sensitization in an urban atopic population without occupational exposure. Respir Med.

[33] Liccardi G, D’Amato G, Antonicelli L, Berra A, Billeri L, Canonica GW, Casino G, Cecchi L, Folletti I, Gani F, Lombardi C, Lo Schiavo M, Meriggi A, Milanese M, Passalacqua G, Pio R, Rolla G, Russo M, Scaccianoce S, Senna GE, Scavalli P, Scichilone N, Sposato B, Siracusa A, Ventura MT (2011). Sensitization to horse allergens in Italy: a multicentre study in urban atopic subjects without occupational exposure. Int Arch Allergy Immunol.

[34] Novembre E, Mori F, Barni S, Pucci N, Rossi ME (2009). Should the skin prick test to horse be included in the standard panel for the diagnosis of respiratory allergy?. J Investig Allergol Clin Immunol.

[35] Liccardi G, Baldi G, Ciccarelli A, Cutajar M, D’Amato M, Gargano D, Giannattasio D, Leone G, Lo Schiavo M, Madonna F, Montera C, Pio A, Russo M, Salzillo A, Stanziola A, D’Amato G, On behalf of Italian Association of Hospital and Territorial Allergologists (AAITO - Campania District, Southern Italy) (2013). Sensitization to rodents (mouse/rat) in urban atopic populations without occupational exposure living in Campania District (Southern Italy). A multicenter study. Multidiscip Respir Med.

[36] Liccardi G, Salzillo A, Sofia M, Piccolo A, Dente B, Russo M, D’Amato M, Stanziola A, D’Amato G (2012). Sensitization to rodents (mouse/rat) in an urban atopic population without occupational exposure living in Naples, Italy. Eur Ann Allergy Clin Immunol.

[37] Liccardi G, Passalacqua G, Salzillo A, Piccolo A, Falagiani P, Russo M (2011). Is sensitization to furry animals an independent allergic phenotype in non-occupationally exposed individuals?. J Investig Allergol Clin Immunol.

[38] Liccardi G, Meriggi A, Russo M, Croce S, Salzillo A, Pignatti P (2015). The risk of sensitization to furry animals in patients already sensitized to cat/dog: A *in vitro* evaluation using molecular-based allergy diagnostics. J Allergy Clin Immunol.

[39] Konradsen JR, Fujisawa T, van Hage M, Hedlin G, Hilger C, Kleine-tebbe J, Matsui EC, Roberts G, Ronmark E, Platts-Mills TAE (2015). Allergy to furry animals: new insights, diagnostic approaches, and challenges. J Allergy Clin Immunol.

[40] Portnoy JM, Kennedy K, Sublett JL, Phipatanakul W, Matsui E, Barnes C (2012). Environmental assessment and exposure control: a practice parameter-furry animals. Ann Allergy Asthma Immunol.

[41] van Hage M, Pauli G (2014). New vaccines for mammalian allergy using molecular approaches. Front Immunol.

[42] Liccardi G, Salzillo A, Cecchi L, D’Amato M, D’Amato G (2012). Is cat keeping the main determinant of new-onset adulthood cat sensitization?. J Allergy Clin Immunol.

[43] Liccardi G, D’Amato M, Pio A, Montera MC, Lo Schiavo M, Sapio C, D’Amato G (2012). Are new pets really responsible for development of new allergies?. Allergol Immunopathol.

[44] Nettis E, Bonifazi F, Bonini S, Di Leo E, Maggi E, Melioli G, Passalacqua G, Senna G, Triggiani M, Vacca A, Canonica GW (2014). Molecular diagnosis and the Italian Board for ISAC. Eur Ann Allergy Clin Immunol.

